# Outpatient clinic specific for end-stage renal disease improves patient survival rate after initiating dialysis

**DOI:** 10.1038/s41598-023-31636-2

**Published:** 2023-04-12

**Authors:** Haruna Fukuzaki, Junichiro Nakata, Shuko Nojiri, Yuki Shimizu, Yuka Shirotani, Takuya Maeda, Toshiki Kano, Maiko Mishiro, Nao Nohara, Hiroaki Io, Yusuke Suzuki

**Affiliations:** 1 Department of Nephrology, Faculty of Medicine, Juntendo University, 2-1-1 Hongo, Bunkyo-ku, Tokyo, 113-8421 Japan; 2 Medical Technology Innovation Center, Juntendo University, Tokyo, Japan; 3 Department of Nephrology, Juntendo University Nerima Hospital, Tokyo, Japan

**Keywords:** Medical research, Nephrology

## Abstract

The importance of a shared decision-making (SDM) approach is widely recognized worldwide. In Japan, hospital accreditation involves the promotion of SDM for patients with end-stage renal disease (ESRD) when considering renal replacement therapy (RRT). This study aimed to clarify the effectiveness and long-term medical benefits of SDM in RRT. Patients with ESRD who underwent dialysis therapy were retrospectively divided into those who visited outpatient clinics specific for ESRD (ESRD clinic) supporting RRT selection with an SDM approach (visited group) and those who did not visit the ESRD clinic (non-visited group). Data of 250 patients (129 in the non-visited group and 121 in the visited group) were analyzed. Mortality was significantly higher in the non-visited group than in the visited group. Not seeing an ESRD specialist was associated with emergent initiation of dialysis and subsequent 1 year mortality. The number of patients who chose peritoneal dialysis as a modality of RRT was significantly larger in the visited group. These findings demonstrate the association between the ESRD clinic, 1 year survival in patients with ESRD after initiating dialysis, and the different RRT modalities. This specific approach in the ESRD clinic may improve the management of patients with ESRD.

## Introduction

The number of patients with end-stage renal disease (ESRD) is increasing worldwide^1,2^. The number of Japanese patients on dialysis reached approximately 340,000 at the end of 2018^3^. In Japan, the number of patients with ESRD per capita and rates of clinical outcomes of dialysis therapy, including survival rate, is one of the highest worldwide^4^. However, some reports have shown that the survival rate within 4 months of dialysis initiation in Japan did not substantially differ from that of other countries^4–6^ and that urgent initiation of dialysis was suggested as one of the contributing factors for mortality.

In Japan, more importance has been given to hemodialysis (HD), whereas only a limited number of patients undergo peritoneal dialysis (PD)^1^. Moreover, the number of renal transplantations is extremely low compared with that in other countries (1800 events per year; 12.5/1,000,000 persons)^7^. This suggests that renal replacement therapy (RRT) in Japan is heavily focused on HD despite it being only a part of RRT. Although all patients with chronic kidney disease (CKD) should be informed about all options for RRT, some patients are only provided with information about HD, and little or no information about PD and renal transplantation is given^8^.

In recent years, patient-centeredness has been attracting attention. Patient-centeredness emphasizes the patient's values and medical point of view when deciding on a treatment plan and evaluating the effectiveness of medical care^9,10^. One of the key components of patient-centeredness is shared decision-making (SDM), which is the process of collaborative deliberation whereby health professionals and patients work together to reach an agreement on preferred healthcare choices from all available treatment options^11,12^. Kidney diseases are often chronic and directly affect patients’ quality of life. Therefore, the SDM approach is inevitable for patient decision-making. In recent years, the importance of SDM for the selection of RRT has been widely recognized in Japan. In fact, health insurance in Japan offers economic incentives for facilities that introduce the SDM approach for RRT since April 2020. Moreover, a new qualification was organized in March 2022 for those who led multi-disciplinary teams for better selection of RRT.

The beneficial effects of SDM for the selection of RRT have been previously reported, including the increased selection of PD and kidney transplantation^13,14^, improved quality of life^15^, and moderation of the estimated glomerular filtration rate (eGFR) slopes before and after SDM^16^. However, the long-term medical benefits derived from SDM for RRT selection in outpatients have not yet been discussed. Therefore, this retrospective study was conducted with a longer follow-up to determine the effectiveness of special management in outpatients specific for ESRD supporting RRT selection with the SDM approach.

## Results

### Characteristics of enrolled participants

**Figure 1:**
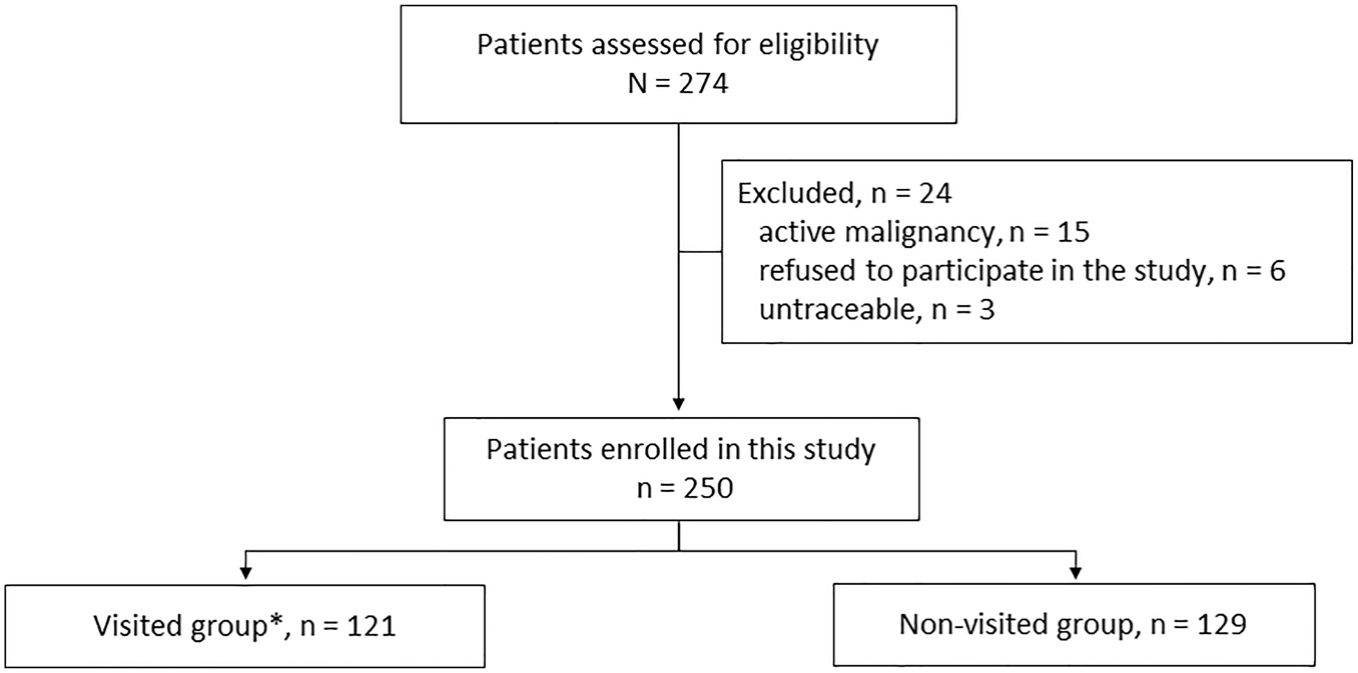
Allocation and course of study participants. RRT, renal replacement therapy; visited group*, those who visited an ESRD clinic and RRT selection with the SDM approach.

During the study period, 274 patients initiated dialysis therapy, including both HD and PD. The following patients were excluded from the study: 15 patients receiving palliative care owing to end-stage malignancy, 6 patients who refused to participate in the study, and 3 untraceable patients (lost to follow-up). Finally, 250 patients were enrolled in the study. Patients were retrospectively divided into those who were seen by the ESRD specialists, called the visited group (n = 121), and those who were not seen by the ESRD specialists, called the non-visited group (n = 129) (Fig. 1). The patients’ characteristics are shown in Table 1. The average eGFR at the first visit to the ESRD clinic in the visited group was 8.58 mL/min/1.73 m^2^.

**Table 1:** Baseline characteristics of the participants.

	Total (n = 250)	Visited group (n = 121)	Non-visited group (n = 129)	*p* value
Age (years)	67.2 ± 13.9	67.3 ± 13.0	67.1 ± 14.8	0.903
≥ 80 years (%, [n])	20.4 (51)	19.0 (23)	21.7 (28)	0.640
Male, sex (%, [n])	64.8 (162)	68.6 (83)	61.2 (79)	0.236
Primary cause of ESRD				
DM (%, [n])	31.6 (79)	31.4 (38)	31.8 (41)	1
CGN (%, [n])	14.8 (37)	14.2 (17)	15.5 (20)	0.859
Nephrosclerosis (%, [n])	17.2 (43)	21.5 (26)	13.2 (17)	0.095
Hereditary (%, [n])	8.8 (22)	13.2 (16)	4.7 (6)	0.024*
Others (%, [n])	20.8 (52)	13.2 (16)	20.2 (26)	0.176
Unknown (%, [n])	10.8 (27)	6.6 (8)	14.7 (19)	0.043*
**CKD stage at first visit to a nephrologist**				
1 (%, [n])	6.8 (17)	3.3 (4)	10.1 (13)	0.043*
2 (%, [n])	4.8 (12)	6.6 (8)	3.1 (4)	0.242
3 (%, [n])	20.8 (52)	25.6 (31)	16.3 (21)	0.086
4 (%, [n])	37.2 (93)	43.0 (52)	31.8 (41)	0.088
5 (%, [n])	30.2 (76)	21.5 (26)	38.8 (50)	0.003*
Emergent initiation of dialysis (%, [n])	51.2 (128)	21.1 (27)	78.9 (101)	< 0.001*
**Past history and complications**				
Hypertension (%, [n])	88.0 (220)	88.4 (107)	87.6 (113)	0.849
DM (%, [n])	43.6 (109)	43.0 (52)	44.2 (57)	0.899
CVD (%, [n])	54.0 (135)	46.3 (56)	61.2 (79)	0.022*
Malignancy (%, [n])	16.8 (42)	19.0 (23)	14.7 (19)	0.400
AKI (%, [n])	11.2 (28)	6.6 (8)	15.5 (20)	0.028*
General anesthesia (%, [n])	33.2 (83)	34.7 (42)	31.8 (41)	0.680
Nephrologist’s care < 6 months (%, [n])	25.2 (63)	8.3 (10)	41.1 (53)	< 0.001*
**Patients’ data at dialysis initiation**				
Body mass index (kg/m^2^)	24.1 ± 4.8	24.7 ± 4.5	23.5 ± 5.1	0.061
Systolic blood pressure (mmHg)	150.1 ± 24.2	150.4 ± 20.8	149.8 ± 27.1	0.839
eGFR (mL/min/1.73 m^2^)	6.6 ± 3.4	5.8 ± 2.3	7.4 ± 4.1	< 0.001*
Hemoglobin (g/dL)	9.5 ± 1.6	9.8 ± 1.5	9.3 ± 1.6	0.005*
Albumin (g/dL)	3.1 ± 0.7	3.4 ± 0.5	2.9 ± 0.7	< 0.001*
C-reactive protein (mg/dL)	1.6 ± 4.3	0.8 ± 2.2	2.5 ± 5.5	0.002*

Data are presented as mean ± standard deviation (range) unless otherwise stated.

ESRD, end-stage renal disease; DM, diabetes mellitus; CGN, chronic glomerular nephropathy; CKD, chronic kidney disease; CVD, cardiovascular disease; AKI, acute kidney injury; eGFR, glomerular filtration rate. *p < 0.05.

### Analysis for a primary outcome

**Figure 2:**
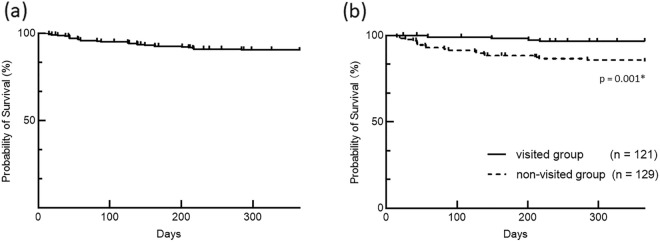
(**a**) Survival rate in all patients. The survival rate after initiation of dialysis was 89.3%. (**b**) Survival rates in the visited and non-visited groups were 96.7% and 84.5%, respectively. The mortality rate was significantly higher in the non-visited group than in the visited group. (p = 0.001*).

The 1-year survival rate after initiation of dialysis was 89.3% (Fig. 2a). Patients in the non-visited group had a significantly higher mortality rate compared with patients in the visited group (*p* = 0.001) (Fig. 2b).

**Figure 3:**
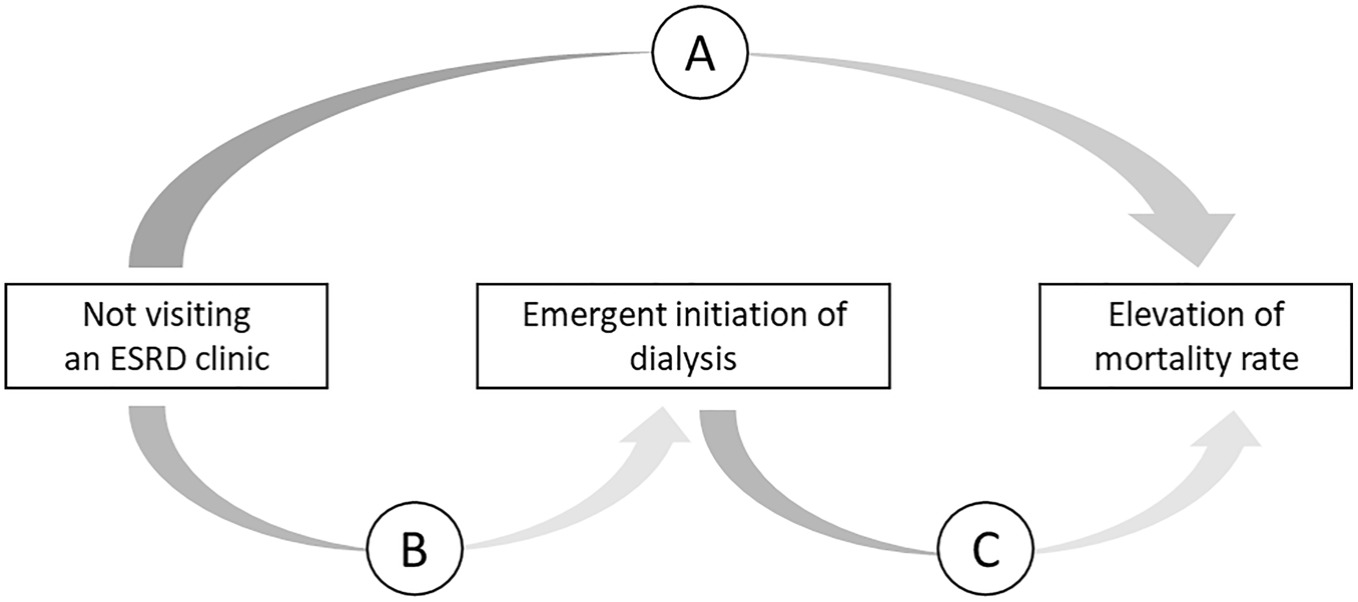
A hypothesis of the relationship between “Not visiting an ESRD clinic,” “Emergent initiation of dialysis,” and “Elevation of mortality rate”.

Previous reports demonstrated that emergent initiation of dialysis significantly impacted mortality. We hypothesized that visiting an ESRD clinic influences emergent initiation of dialysis, which affects the survival rate of patients with ESRD (Fig. 3). As shown in Table 2, the univariable and multivariable Cox proportional hazard model was applied to evaluate the relation between visiting an ESRD clinic and mortality rate (labeled “A” in Fig. 3). The univariable analysis showed that not visiting an ESRD clinic, cardiovascular disease, and the presence of acute kidney injury (AKI) were risk factors for death within 1 year. Multivariable analysis showed that not being examined by ESRD specialists led to a 3.892-fold higher risk of death within a year (HR = 3.892: 95% CI = 1.300–11.645; *p* = 0.015).

**Table 2:** Analysis of the relationship between not visiting an ESRD clinic and mortality rate (A in Fig. 3).

	Univariable analysis			Multivariable analysis		
	HR	95% CI	*p* value	HR	95% CI	*p* value
Not visiting an ESRD clinic	5.045	1.724–14.760	0.003*	3.892	1.300–11.645	0.015*
Male (vs. Female)	0.743	0.330–1.674	0.474			
DM (vs. without DM)	0.760	0.333–1.737	0.515			
CVD (vs. without CVD)	2.637	1.047–6.642	0.039*	2.026	0.794–5.171	0.139
AKI (vs. without AKI)	4.568	1.954–10.681	< 0.001*	3.540	1.497–8.374	0.004*
General anesthesia	1.510	0.671–3.400	0.319			
Age (per 1 year increase)	1.027	0.995–1.061	0.099			

HR, hazard ratio; CI, confidence interval; ESRD, end-stage renal disease; DM diabetes mellitus; CVD, cardiovascular disease; AKI, acute kidney injury. *p value < 0.05.

As shown in Table 3, simple and multiple logistic regression analyses were applied to determine the relationship between visiting an ESRD clinic and emergent initiation of dialysis (labeled “B” in Fig. 3). In the multivariable analysis, not visiting an ESRD clinic led to a 9.963-fold increasing risk of emergent initiation of dialysis (OR = 9.963; 95% CI = 5.056–18.376; *p* < 0.001).

**Table 3:** Analysis of the relationship between not visiting an ESRD clinic and emergent initiation of dialysis (B in Fig. 3).

	Univariable analysis			Multivariable analysis		
	OR	95% CI	*p* value	OR	95% CI	*p* value
Not visiting an ESRD clinic	12.558	6.901–22.851	< 0.001*	9.963	5.056–18.376	< 0.001*
Male (vs. Female)	0.529	0.312–0.898	0.018*	0.422	0.212–0.837	0.013*
DM (vs. without DM)	1.231	0.746–2.032	0.415			
CVD (vs. without CVD)	2.658	1.592–4.436	< 0.001*	2.781	1.461–5.295	0.001*
AKI (vs. without AKI)	2.188	0.949–5.046	0.066			
General anesthesia	0.898	0.530–1.520	0.687			
Age (per 1-year increase)	1.006	0.988–1.024	0.536			
Nephrologist’s care < 6 months	6.071	3.036–12.141	< 0.001*	2.905	1.291–6.536	0.010*

OR, odds ratio. *p value < 0.05.

As shown in Table 4, the univariable and multivariable Cox proportional hazard model was again applied to evaluate the relation between emergent initiation of dialysis and mortality rate (labeled “C” in Fig. 3). Additionally, we performed multivariable analysis by analyzing the patients’ comorbidities (Model 1) and parameters related to the patients’ condition (Model 2) on initiation of dialysis. In the multivariable analysis, emergent initiation of dialysis led to a 5.677-fold (HR = 5.677; 95% CI = 1.635–19.496; *p* = 0.005) and 4.564-fold (HR = 4.564; 95% CI = 1.259–16.545; *p* = 0.020) increase in the risk of death within one year in models 1 and 2, respectively.

**Table 4:** Analysis of the relationship between emergent initiation of dialysis and mortality rate (C in Fig. 3).

	Univariable analysis			Multivariable analysis (model 1)			Multivariable analysis (model 2)		
	HR	95% CI	*p* value	HR	95% CI	*p* value	HR	95% CI	*p* value
Emergent initiation (vs. planned)	7.231	2.157–24.246	0.001*	5.677	1.653–19.496	0.005*	4.564	1.259–16.545	0.020*
Male (vs. Female)	0.743	0.330–1.674	0.474						
DM (vs. without DM)	0.760	0.333–1.737	0.515						
CVD (vs. without CVD)	2.637	1.047–6.642	0.039*	1.773	0.692–4.539	0.232			
AKI (vs. without AKI)	4.568	1.954–10.681	< 0.001*	3.736	1.591–8.773	0.002*			
General anesthesia	1.510	0.671–3.400	0.319						
Age (per 1 year increase)	1.027	0.995–1.061	0.099						
Alb (per 1 mg/dL increase)	0.424	0.263–0.684	< 0.001*				0.615	0.346–1.093	0.097
Hb (per 1 g/dL increase)	0.910	0.707–1.171	0.463						
CRP (per 1 mg/dL increase)	1.050	1.012–1.090	0.009*				1.017	0.963–1.074	0.539
SBP < 130 mmHg	5.205	2.330–11.625	< 0.001*				4.825	2.155–10.803	< 0.001*

Alb, albumin: Hb, hemoglobin; CRP, C-reactive protein; SBP, systolic blood pressure. *p value < 0.05.

The results shown in Tables 2, 3, and 4 indicate that not being examined by ESRD specialists had a significant impact on emergent initiation of dialysis, which subsequently led to higher mortality within 1 year.

Additionally, a mediation analysis was conducted to quantify the direct and indirect effects on mortality within a year of visiting an ESRD clinic through emergent initiation of dialysis (Supplementary Material).

### Analyses of secondary outcomes

As shown in Table 5, significantly more patients chose PD when they underwent RRT modality choice using SDM as an outpatient. Medical costs were smaller and hospitalizations were shorter in this group.

**Table 5:** Analyses of secondary outcomes.

	Total (n = 250)	Visited group (n = 121)	Non-visited group (n = 129)	*p* value
RRT modality choice				
PD (%, [n])	10.0 (25)	15.7 (19)	4.7 (6)	< 0.001*
Kidney transplantation within a year after initiation of dialysis (%, [n])	1.6 (4)	1.7 (2)	1.6 (2)	1
Emergent initiation of dialysis, medical costs, and hospitalization				
Emergent initiation (%, [n])	51.2 (128)	22.3 (27)	78.3 (101)	< 0.001*
Medical costs (*10^4^JPY)	246 ± 232.7	144.5 ± 108.3	341.7 ± 274.5	< 0.001*
Hospitalization (days)	38.7 ± 29.0	22.7 ± 13.0	53.7 ± 31.7	< 0.001*

Data are presented as mean ± standard deviation (range) unless otherwise stated.

PD, peritoneal dialysis. *p value < 0.05.

## Discussion

This was the first study to investigate the long-term effects of outpatient specific for ESRD management, including SDM for selecting the RRT modality, on patient outcomes. Our results demonstrate that patients who were not seen by ESRD specialists (non-visited group) had a significantly higher rate of emergent initiation of dialysis, which subsequently led to a higher mortality rate. In addition, the relationship between specialized management of patients with ESRD, emergent initiation of dialysis, and survival rate were confirmed.

As we previously reported^17^, emergent initiation of dialysis is related to increased mortality^18–20^. Patients who undergo urgent initiation of dialysis often require rapid care and the poor general condition at that time of dialysis worsens the survival rate. Risk factors associated with emergent-start dialysis include AKI, diabetes mellitus, heart failure, and late nephrologist referral. In clinical practice, nephrologists should try to reduce the risk of urgent initiation of dialysis and improve the prognosis of patients who require an emergent-start of chronic dialysis. Our study demonstrated that visiting an ESRD clinic and RRT modality selection with SDM for the management of long-term dialysis therapy reduces emergent initiation of dialysis. We hypothesize that the “specificity” of this clinic is different from the usual visit of nephrologists.

First, visiting an ESRD clinic is a chance for patients with CKD to accept their advanced CKD condition. Finkelstein et al.^21^ previously reported that a large proportion of patients with CKD (stage 3–5) have limited knowledge on kidney diseases and are unaware of CKD progression ^22,23^. Remarkably, the level of patient knowledge concerning CKD is limited even in those who regularly visit nephrologists^21^. In starting a conversation about the RRT modality, the reason they were referred to the special clinic will be discussed. The chance to correctly comprehend the status of their disease leads to constructive discussion about the management of ESRD and consideration of the RRT modality.

Second, the ESRD clinic focuses on providing necessary and sufficient information and discussing RRT with patients and their families. Although it seems natural for individuals with kidney failure who have not started RRT to go through this process, patients are often not given enough information to make an informed choice or choices are made for the patients^8^. Moreover, 46.8% of HD patients interviewed in the Empowering Patients on Choices for Renal Replacement Therapy (EPOCH-RRT) study thought that undergoing HD as a dialysis modality had not been their choice, whereas this was only reported by 2.6% of patients who underwent PD^24^. This result suggests that individuals who received limited information and opportunities to make an informed choice were unlikely to choose PD as a dialysis modality. Moreover, delivering information about RRT may be biased according to the knowledge, medical experience, and expertise of the nephrologists and the facilities^8,25^. Some patients have little or no information about PD or renal transplantation. These findings suggest that insufficient and prejudiced information about RRT is prevalent for patients with kidney dysfunction due to the convenience of the healthcare professionals.

Third, a visit to the ESRD clinic is a precious opportunity for healthcare professionals to understand not only the patient’s CKD conditions and comorbidities but also social factors that may influence outcomes. Fages et al*.*^19^ suggested that both living alone and low health literacy were significantly associated with urgent-start dialysis, even if patients were referred to nephrologists early. When considering RRT modalities, the surrounding factors of the patient are inseparable from their selections, including occupations, family members, those whom the patients share their lives with, the degree to which the patients need long-term care or support, the degree of cognitive function, the type of health insurance, and patients’ values and preferences. In particular, health literacy has not been assessed in routine nephrology practice but should be given much more attention because of the complexity of renal failure management^19,26^.

SDM has been described as a process of collaborative deliberation whereby health professionals and patients work together to reach an agreement on preferred healthcare choices from all available treatment options^11^. These choices integrate the best evidence available with the patients’ values and preferences to promote high-quality health care decisions^27^. The SDM approach has a significant influence on preventing urgent-start of dialysis.

Regarding secondary outcomes, our results agree with previous reports showing that visiting an ESRD clinic and RRT selection with the SDM approach increase the rate of patients selecting PD. Two patients who underwent kidney transplantation within a year after the initiation of dialysis in the non-visited group experienced CKD educational admission and underwent RRT modality choice during hospitalization. These results revealed that the ESRD clinic contributed to the diversity of RRT modality decisions.

There are several limitations to this study. First, the study population was limited to those who started dialysis therapy at Juntendo University Hospital. The number of renal transplantations is extremely low in Japan (1800 events per year; 12.5/1,000,000 persons) as previously mentioned^7^, suggesting the limited number of facilities for transplantation in Japan. Although there were some patients with ESRD those who visited this ESRD clinic subsequently underwent preemptive kidney transplantation, they had been referred to other facilities until when renal transplantation first started at the Juntendo University Hospital in 2020. It is necessary to include ESRD patients who underwent kidney transplantation into study population to correctly evaluate the effectiveness of the clinic. Second, we could not collect data on certain confounders due to the lack of information in our study. Factors such as polypharmacy, living alone, low health literacy, poor predialysis care, nonattendance at dialysis information sessions, surgical delays in vascular access, and lack of patient education programs^19,28–32^ are considered risk factors for unplanned dialysis, and these factors could cause issues related to the unmeasured confounders of the emergent initiation of dialysis in this study. For example, Victor Fages et al. reported that the adjusted odds ratios for urgent-start dialysis were 2.14 (1.47–4.57), 2.22 (1.28–3.84), and 2.14 (1.17–3.90) for polypharmacy, living alone, and low health literacy, respectively^19^. We recognize that these unmeasured confounders are a potential source of bias in our cohort study; therefore, future studies including these variables should be conducted to provide an accurate estimate. Third, our study did not indicate the suitable time for referring patients with CKD to the ESRD clinic. Patients were introduced to ESRD specialists during their regular visits to attending nephrologists, and the decision to refer the patients to the ESRD clinic was made by the nephrologist. The average eGFR at the first visit to an ESRD clinic in the visited group was 8.58 mL/min/1.73 m^2^; notably, 94.2% visits to the ESRD clinic were made after CKD advanced to stage 5. Although there is no global consensus regarding the time to start SDM on RRT, our study may help in emphasizing the importance of “starting” SDM, regardless of the advancement in the stage of CKD.

In conclusion, being seen by ESRD specialists and undergoing the SDM approach for RRT modality selection has a significant impact on preventing emergent initiation of dialysis, which subsequently leads to better 1-year survival. This approach may reduce the risk of urgent-start dialysis and improve the management of patients with ESRD.

## Methods

We conducted a retrospective single-institution cohort study to evaluate the efficacy of special outpatient management for ESRD and RRT selection with the SDM approach. The study protocol was approved by the Ethics Committee of Juntendo University Hospital, Tokyo, Japan (approval number: 17-175). All procedures involving human participants were performed in accordance with the ethical standards of the institutional and/or national research committee and the 1964 Helsinki declaration and its later amendments or comparable ethical standards. All participants provided written informed consent before enrollment. The study was registered with the University Hospital Medical Information Network Clinical Trials Registry on 01/09/2019 (UMIN000039224).

The primary outcome of this study was the 1-year survival rate following the initiation of dialysis, and the secondary outcomes included the diversity of RRT modality choices, medical costs, and the hospitalization period.

### Participants

The inclusion criteria for this study included the commencement of dialysis therapy, both HD and PD, from January 2018 to December 2020 at Juntendo University Hospital, Tokyo, Japan. Meanwhile, patients were excluded if they were receiving palliative care owing to end-stage malignancy or refused to participate in the study.

### Definition of an ESRD clinic

We have had an ESRD clinic since April 2017 in the Department of Nephrology, Juntendo University Hospital. The nephrologists and nurses in this clinic are well-acquainted with the treatment of renal failure, including dialysis therapy (PD and HD) and kidney transplantation. These specialists provide patients and their families with necessary and sufficient information about RRT and interact with the patients while choosing the best treatment modality using the SDM approach. Explanations of RRT are provided using a document entitled “Renal Failure; Treatment Selection and Practice” (https://jsn.or.jp/jsn_new/iryou/kaiin/free/primers/pdf/2022allpage.pdf), which has been compiled by the Japanese Society of Nephrology, the Japanese Society for Dialysis Therapy, the Japanese Society for Transplantation, the Japanese Society for Clinical Renal Transplantation, and the Japanese Society for Peritoneal Dialysis. In addition, upon visiting the clinic, the patients are asked to complete the “Kidney Disease; Choosing the Best Treatment for You” (https://www.ckdsdm.jp/document/booklet/images/sdm.pdf) form, provided by the Japan Shared Decision Making Collaborative for Chronic Kidney Disease, in advance to ensure that they understand the disease and its treatment and to help in organizing their thoughts. An assessment for necessary long-term access to dialysis therapy is subsequently made, including vascular access (VA) creation and PD catheter insertion. Patients are introduced to ESRD specialists during their regular visits, and decisions to refer the clinic are left to each attending nephrologist.

### Definitions of emergent initiation of dialysis, medical costs, and period of hospitalization

Emergent-start dialysis was defined as cases in which long-term VA access was not in place for HD patients and PD catheters were not placed in patients with PD before initiation of dialysis therapy, as previously reported^17^. For medical costs and the period of hospitalization for preparing and initiating dialysis therapy, hospitalization for the creation of VA or stepwise initiation of PD using the Moncrief and Popovich techniques were included in this study, even without the initiation of dialysis.

### Follow-up

Patient information was collected from the satellite dialysis clinics after discharge from our hospital, including the date and cause of death.

### Statistical analysis

The clinical characteristics of participants are presented as means ± standard deviations for continuous variables and counts and proportions for categorical variables. The 1 year survival rate was analyzed using the Kaplan–Meier technique. The difference in survival rates between the two groups was examined using a log-rank test.

Univariable and multivariable Cox proportional hazard models were applied to evaluate risk factors for survival, and hazard ratios (HRs) and 95% confidence intervals (CIs) are presented. The relationship between emergent dialysis initiation and risk factors was assessed by simple and multiple logistic regression analyses. Clinical variables for univariable and multivariable analyses were selected based on prior knowledge about the variables. The statistically significant variables from the univariable analyses were included in the multivariable analysis.

Statistical analyses were performed using the SAS software (version 9.4, SAS Institute, Cary, NC). A *p* value of < 0.05 was considered statistically significant.

## Supplementary Information

Supplementary Information.

## Data Availability

The datasets analyzed in this study are available from the corresponding author on reasonable request.
