# Supplementary material for: Outpatient clinic specific for end-stage renal disease improves patient survival rate after initiating dialysis
Source: Sci Rep. 2023 Apr 12;13:5991. doi: 10.1038/s41598-023-31636-2 (PMC10097859; doi:10.1038/s41598-023-31636-2)
Supplement: Supplementary file 1 — Supplementary Information. [file 41598_2023_31636_MOESM1_ESM.docx]

**Supplementary material**

**Methods**

We conducted a mediation analysis to quantify the direct and indirect effects on mortality of an ESRD clinic through emergent initiation of dialysis. The causal mediation analysis processes are framed in terms of intermediate variables between an independent variable and a dependent variable, where *t* is the independent variable, *Y* is the dependent variable, and *M* is the mediator variable that is supposed to transmit the causal effect of *t* to *Y*. The total effect of *t* on *Y* is referred to as the total effect (TE), and that effect is partitioned into a combination of a direct effect (DE) of *t* on *Y* and an indirect effect (IE) of *t* on *Y* that is transmitted through *M* ^1^. We defined the causal mediation effect or indirect effect for each unit *i*:

$\delta_{i}\left( t \right)\equiv Y_{i}\left( t,M_{i}\left( 1 \right) \right)-Y_{i}\left( t,M_{i}\left( 0 \right) \right)$, (1)

for $t=0,1$.

We considered “mortality (death or being alive) within a year” as a binary outcome, and we defined “emergent initiation of dialysis” as a mediator and “not visiting an ESRD clinic” as an exposure. The causal mediation effect represents the indirect effect of the exposure (not visiting an ESRD clinic) on the outcome (death within 1 year) through the mediating variable (emergent initiation of dialysis), whereas the direct effect is the effect of the exposure (not visiting an ESRD clinic) on the outcome (death within 1 year) that is independent of the mediator (emergent initiation of dialysis). The total effect is the sum of the indirect and direct effects.

$Y_{i}$ denotes the outcome (death) for the patient *i*; *t* is the exposure (visiting an ESRD clinic) status; *t* = 1 denotes no visiting an ESRD clinic; and *t* = 0 denotes visiting an ESRD clinic. This determines what change would occur in the outcome if one changed the mediator value (emergency initiation of dialysis) that would be realized under the presence of mediator *Mi* (1) to the value that would be observed under the absence of mediator *Mi* (0) while keeping the exposure status constant. If this has no effect on mortality, the causal mediation effect is zero.

In addition, the direct effect of the treatment on each unit is as follows:

$\zeta_{i}(t)\equiv Y_{i}(1,M_{i}\left( t \right))-Y_{i}(0,M_{i}\left( t \right))$, (2)

for t = 0,1.

The total effect of the treatment can be conceived as causal mediation and direct effects as follows:

$\tau_{i}\equiv Y_{i}(1,M_{i}\left( 1 \right))-Y_{i}\left( 0,M_{i}\left( 0 \right) \right)=\frac{1}{2}\sum_{i=0}^{1} \left\{ \delta_{i}\left( t \right)+\zeta_{i}(t) \right\}$.

Depending on the level of *t*, we have:

$$\delta_{i}\left( 1 \right)\mathbb{=E[}Y_{i}(1,M_{i}\left( 1 \right)-Y_{i}(1,M_{i}\left( 0 \right))]$$

$\delta_{i}\left( 0 \right)\mathbb{=E[}Y_{i}(0,M_{i}\left( 1 \right)-Y_{i}(0,M_{i}\left( 0 \right))]$.

The average causal mediation effect and total effects can be defined as follows:

$$\bar{\zeta}(t)\equiv\mathbb{E(}Y_{i}\left( 1,M_{i}\left( t \right) \right)-Y_{i}\left( 0,M_{i}\left( t \right) \right))$$

and

$$\bar{\tau}\left( t \right)\mathbb{\equiv E}\left( Y_{i}\left( 1,M_{i}\left( 1 \right) \right)-Y_{i}\left( 0,M_{i}\left( 0 \right) \right) \right)=\frac{1}{2}\sum_{t=0}^{1} \left\{ \bar{\delta}\left( t \right)+\bar{\zeta}\left( t \right) \right\},$$

respectively.

Under the assumption of no interaction, the average causal mediation and average direct effects sum to the average total effects. To examine whether a visit to an ESRD clinic affects the mortality intermediated by the emergency initiation of dialysis, we conducted two mediation analyses based on the logistic regression model. We used the R mediation package (<https://cran.r-project.org/web/packages/mediation/index.html>). A p value of <0.05 was considered statistically significant.

**Results**

First, we conducted a mediation analysis without interaction between the terms “not visiting an ESRD clinic” and “emergent initiation of dialysis.” The average direct effect of not visiting an ESRD clinic was not a significant predictor of mortality (average direct effect = −0.05, p = 0.26). With emergency initiation of dialysis, the relationship between not visiting an ESRD clinic and mortality was also not significant (average causal mediation effect = −0.04, p = 0.06) (Supplementary Tables 1-1, 1-2, and 2; Figures 1 and 2).

Subsequently, the mediation analysis of the interaction between the terms “not visiting an ESRD clinic” and “emergent initiation of dialysis” was conducted. The average direct effect of not visiting an ESRD clinic was not a significant predictor of mortality (average direct effect = −0.05, p = 0.24). With emergency initiation of dialysis, the relationship between not visiting an ESRD clinic and mortality was also not significant (average causal mediation effect = −0.04, p = 0.06). (Supplementary Tables 3-1, 3-2, and 4; Figures 3 and 4). Although neither the direct nor indirect effect of an ESRD clinic through emergent initiation of dialysis on mortality was clarified, it was certain that the total effect of not visiting an ESRD clinic has a total effect on death within a year.

**Reference**

1 Imai, K., Keele, L. & Tingley, D. A general approach to causal mediation analysis. *Psychol Methods* **15**, 309-334, doi:10.1037/a0020761 (2010).

**Supplementary Tables and Figure****s**

**Supplementary Table 1-1.** **Multivariable logistic model of mortality (no interaction term)**

| Effects | OR | 95% CI | p value |
| --- | --- | --- | --- |
| Not visiting an ESRD clinic | 0.46 | 0.11, 1.58 | 0.2 |
| Emergent initiation of dialysis (vs. planned) | 0.23 | 0.05, 0.87 | 0.045 |
| Male (vs. Female) | 1.18 | 0.45, 3.03 | 0.7 |
| DM (vs. without DM) | 1.26 | 0.49, 3.40 | 0.6 |
| CVD (vs. without CVD) | 0.62 | 0.23, 1.75 | 0.4 |
| AKI (vs. without AKI) | 0.23 | 0.08, 0.69 | 0.008 |
| General anesthesia | 1.13 | 0.41, 3.32 | 0.8 |
| Age (per 1-year increase) | 0.98 | 0.94, 1.01 | 0.2 |

OR, odds ratio; CI, confidence interval; ESRD, end-stage renal disease; DM diabetes mellitus; CVD, cardiovascular disease; AKI, acute kidney injury.

**Supplementary Table 1-2. Multivariable logistic model** **of mortality (no interaction term)**

| Effects | OR | 95% CI | p value |
| --- | --- | --- | --- |
| Not visiting an ESRD clinic | 9.69 | 5.21, 18.6 | <0.001 |
| Male (vs. Female) | 0.47 | 0.2, 0.91 | 0.026 |
| DM (vs. without DM) | 1.15 | 0.60, 2.20 | 0.7 |
| CVD (vs. without CVD) | 1.39 | 0.6, 2.94 | 0.4 |
| AKI (vs. without AKI) | 1.19 | 0.40, 3.65 | 0.8 |
| General anesthesia | 0.86 | 0.43, 1.72 | 0.7 |
| Age (per 1-year increase) | 1.01 | 0.99, 1.04 | 0.2 |
| Nephrologist’s care <6 months | 3.20 | 1.41, 7.61 | 0.007 |

OR, odds ratio; CI, confidence interval; ESRD, end-stage renal disease; DM diabetes mellitus; CVD, cardiovascular disease; AKI, acute kidney injury.

**Supplementary Table 2. Effects of visiting an ESRD clinic (or not visiting an ESRD clinic) on mortality determined by causal mediation analysis (no interaction term)**

|  | Estimate | 95% CI | p value |
| --- | --- | --- | --- |
| ACME (visiting an ESRD clinic) | −0.03 | −0.09, 0 | 0.06 |
| ACME (not visiting an ESRD clinic) | −0.05 | −0.1, 0 | 0.06 |
| ADE (visiting an ESRD clinic) | −0.04 | −0.13, 0.03 | 0.26 |
| ADE (not visiting an ESRD clinic) | −0.06 | −0.16, 0.06 | 0.26 |
| Total effect | −0.09 | −0.17, −0.01 | 0.03 |
| Proportion mediated (visiting an ESRD clinic) | 0.29 | −0.06, 2.59 | 0.09 |
| Proportion mediated (not visiting an ESRD clinic) | 0.56 | −0.13, 1.8 | 0.09 |
| ACME (average) | −0.04 | −0.09, 0 | 0.06 |
| ADE (average) | −0.05 | −0.14, 0.04 | 0.26 |
| Proportion mediated (average) | 0.42 | −0.1, 2.2 | 0.09 |

Interaction term between not visiting an ESRD clinic and emergent initiation of dialysis was not included. ACME; Average causal mediation effect, ADE; Average direct effect.

**Supplementary Table 3-1.** **Multivariable logistic model of mortality (including interaction term)**

| Effects | OR | 95% CI | p value |
| --- | --- | --- | --- |
| Not visiting an ESRD clinic | 0.60 | 0.05, 13.4 | 0.7 |
| Emergent initiation of dialysis (vs. planned) | 0.28 | 0.03, 2.59 | 0.2 |
| Male (vs. Female) | 1.18 | 0.45, 3.04 | 0.7 |
| DM (vs. without DM) | 1.26 | 0.49, 3.41 | 0.6 |
| CVD (vs. without CVD) | 0.61 | 0.22, 1.73 | 0.3 |
| AKI (vs. without AKI) | 0.23 | 0.08, 0.70 | 0.008 |
| General anesthesia | 1.13 | 0.41, 3.36 | 0.8 |
| Age (per 1-year increase) | 0.98 | 0.94, 1.01 | 0.2 |
| Not visiting an ESRD clinic mediated by emergent initiation of dialysis | 0.69 | 0.02, 11.8 | 0.8 |

OR, odds ratio; CI, confidence interval; ESRD, end-stage renal disease; DM diabetes mellitus; CVD, cardiovascular disease; AKI, acute kidney injury.

**Supplementary Table 3-2.** **Multivariable logistic model of** **mortality (including interaction term)**

| Effects | OR | 95% CI | p value |
| --- | --- | --- | --- |
| Not visiting an ESRD clinic | 9.69 | 5.21, 18.6 | <0.001 |
| Male (vs. Female) | 0.47 | 0.24, 0.91 | 0.026 |
| DM (vs. without DM) | 1.15 | 0.60, 2.20 | 0.7 |
| CVD (vs. without CVD) | 1.39 | 0.66, 2.94 | 0.4 |
| AKI (vs. without AKI) | 1.19 | 0.40, 3.65 | 0.8 |
| General anesthesia | 0.86 | 0.43, 1.72 | 0.7 |
| Age (per 1-year increase) | 1.01 | 0.99, 1.04 | 0.2 |
| Nephrologist’s care < 6 months | 3.20 | 1.41, 7.61 | 0.007 |

OR, odds ratio; CI, confidence interval; ESRD, end-stage renal disease; DM diabetes mellitus; CVD, cardiovascular disease; AKI, acute kidney injury.

**Supplementary Table 4.** **Effects of visiting an ESRD clinic (or not visiting an ESRD clinic) on mortality determined by causal mediation analysis (including interaction term)**

|  | Estimate | 95% CI | p value |
| --- | --- | --- | --- |
| ACME (visiting an ESRD clinic) | −0.03 | −0.11, 0.01 | 0.18 |
| ACME (not visiting an ESRD clinic) | −0.05 | −0.11, 0.02 | 0.12 |
| ADE (visiting an ESRD clinic) | −0.04 | −0.16, 0.04 | 0.29 |
| ADE (not visiting an ESRD clinic) | −0.06 | −0.18, 0.07 | 0.26 |
| Total effect | −0.1 | −0.18, −0.01 | 0.03 |
| Proportion mediated (visiting an ESRD clinic) | 0.27 | −0.18, 3.18 | 0.2 |
| Proportion mediated (not visiting an ESRD clinic) | 0.62 | −0.49, 1.95 | 0.15 |
| ACME (average) | −0.04 | −0.09, 0 | 0.06 |
| ADE (average) | −0.05 | −0.15, 0.05 | 0.24 |
| Proportion mediated (average) | 0.45 | −0.09, 2.41 | 0.09 |

The interaction term between not visiting an ESRD clinic and emergent initiation of dialysis was not included. ACME; Average causal mediation effect, ADE; Average direct effect.

**Supplementary Figure 1. Mediation analysis without interaction term between -not visiting an ESRD clinic- and -emergent initiation of dialysis-**

**
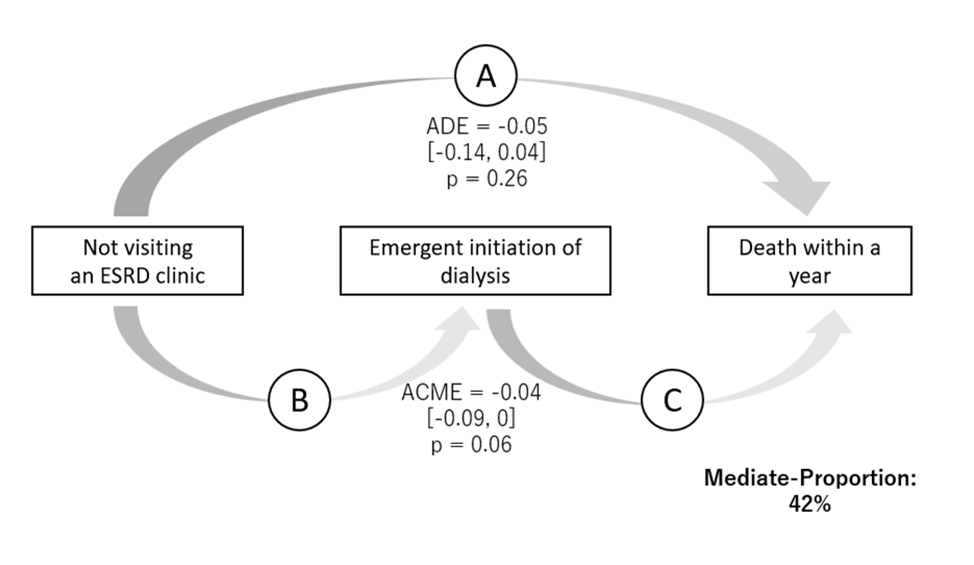
**

**Supplementary Figure 2. Graphical summary of causal mediation analysis results for the effect of not visiting an ESRD clinic**


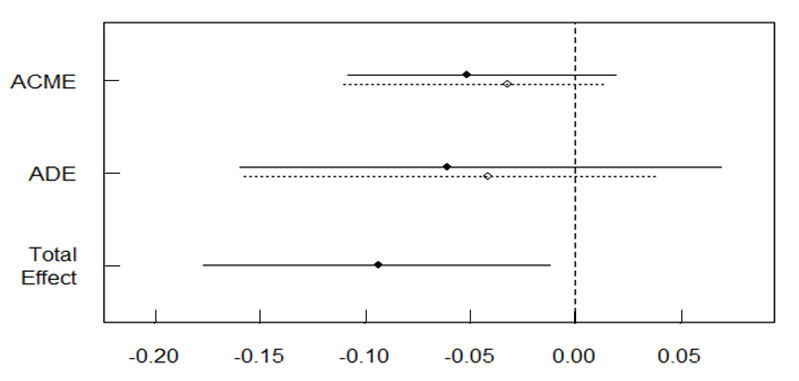


The X-axis represents the effect value; each dot represents the point estimate; and each line represents the confidence interval. The dotted vertical line indicates the absence of an effect of antibodies on ascension.

**Supplementary Figure 3. Mediation analysis with interaction term between -not visiting an ESRD clinic- and -emergent initiation of dialysis-**

**
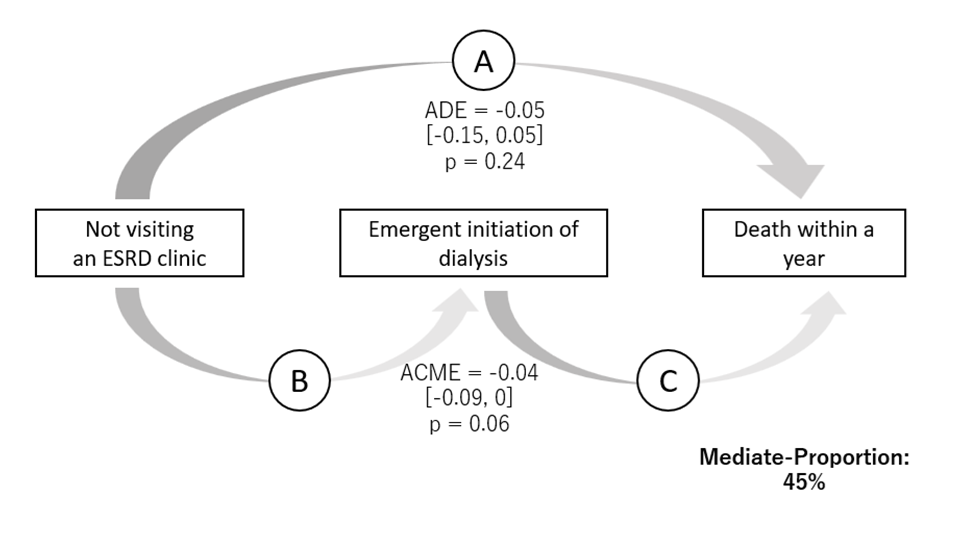
**

**Supplementary Figure 4. Graphical summary of causal mediation analysis results for the effect of not visiting an ESRD clinic**

**
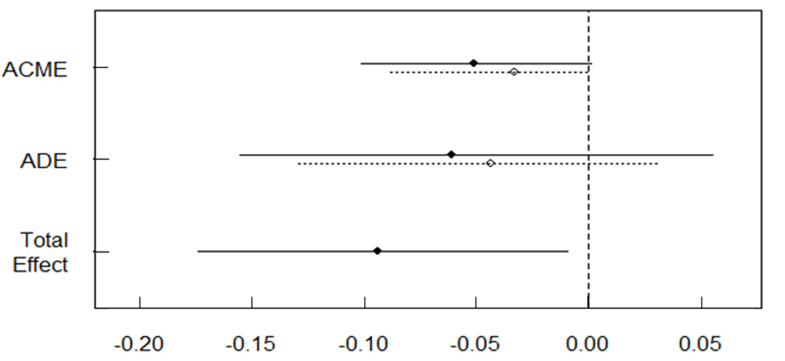
**

The X-axis represents the effect value; each dot represents the point estimate; and each line represents the confidence interval. The dotted vertical line indicates the absence of an effect of antibodies on ascension.
